# P-1864. Early Combination Therapy with Isavuconazole and Immune Checkpoint Inhibitors Improves Infection Outcomes and Alleviates Paralyzed Antifungal Host Immunity in Neutropenic Mice with Invasive Pulmonary Mucormycosis

**DOI:** 10.1093/ofid/ofae631.2025

**Published:** 2025-01-29

**Authors:** Sebastian Wurster, Nathaniel D Albert, Dimitrios P Kontoyiannis

**Affiliations:** The University of Texas MD Anderson Cancer Center, Houston, Texas; The University of Texas MD Anderson Cancer Center, Houston, Texas; The University of Texas MD Anderson Cancer Center, Houston, Texas

## Abstract

**Background:**

Invasive pulmonary mucormycosis (IPM) is a severe opportunistic mold infection whose outcome is predominantly host-driven. Preclinical studies and several clinical case reports suggested a benefit of adjunct immunotherapy with immune checkpoint inhibitors (ICIs). However, the mechanisms of IPM-associated pulmonary immune paralysis and its mitigation by ICIs require further study.

**Figure:**
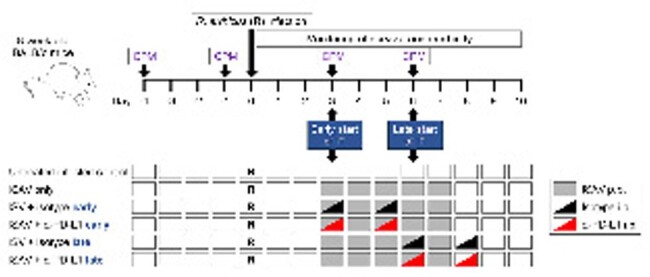

Timeline of experimental interventions. Abbreviations: CPM = cyclophosphamide, i.p. = intra-peritoneally, ISAV = isavuconazonium sulfate, IT = immunotherapy, PD-L1 = Programmed Death Ligand 1, p.o. = per os (oral gavage).

**Methods:**

Eight-week-old cyclophosphamide-immunosuppressed BALB/c mice were infected intranasally with 50,000 *Rhizopus arrhizus* spores. The Mucorales-active triazole isavuconazonium sulfate (ISAV) was initiated on day (d) 3 post-infection (2×64 mg/kg/d by oral gavage). Additionally, mice received 2×0.25 mg/kg anti (α)-PD-L1 or a non-targeting isotype antibody either on d3+5 (early) or d6+8 (late) (Fig. 1). Infection severity was scored using the murine sepsis score (MSS; 0 = healthy – 3 = moribund, 4 = dead). nCounter-based transcriptomics and Ingenuity pathway enrichment analysis were performed on lung tissue homogenates.

**Figure:**
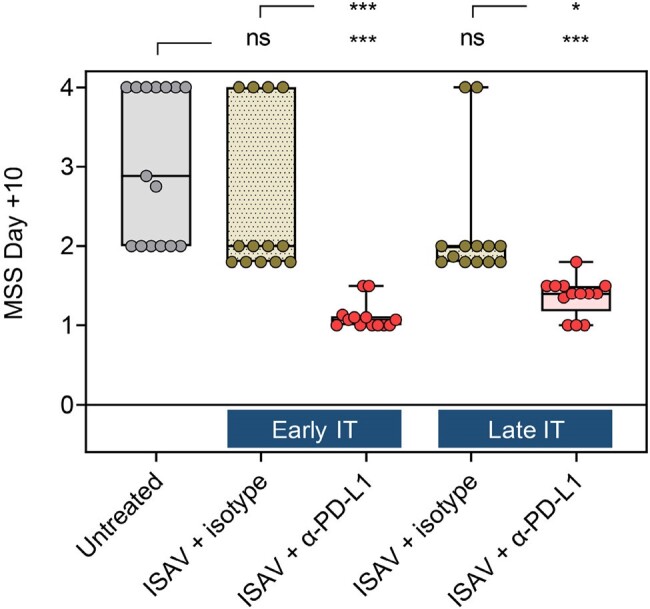

Murine sepsis scores (MSS, 0 = healthy – 3 = moribund, 4 = dead) of immunosuppressed mice with invasive pulmonary mucormycosis according to the treatment arm. Kruskal-Wallis test with Dunn’s post-test. * p<0.05, *** p<0.001. Abbreviations: ISAV = isavuconazonium sulfate, IT = immunotherapy, ns = not significant, PD-L1 = Programmed Death Ligand 1.

**Results:**

Untreated neutropenic mice developed severe IPM (median day-10 MSS: 2.9, Fig. 2). Pulmonary transcriptomics at d4 revealed strong innate immune activation and induction of type-1 (Th1) and type-17 (Th17) T-helper-cell responses. However, by d7, proinflammatory signals had markedly decreased and nCounter analysis revealed impaired intercellular crosstalk and T-cell signaling (Fig. 3). Compared to ISAV + isotype (median d10 MSS, 2.0), early and late adjunct α-PD-L1 therapy improved median d10 MSS to 1.0 (p < 0.001) and 1.4 (p = 0.01), respectively (Fig. 2). While ISAV delayed the tipping point from immune activation to full-blown immune paralysis, early adjunct α-PD-L1 therapy elicited significantly stronger dendritic cell and classical (M1) macrophage signaling, activation of key cytokine pathways (e.g., IL-1, IL-2, IL-12), reinvigoration of Th1 and Th17 signaling, and suppression of IPM-induced co-inhibitory checkpoint pathways than both ISAV + isotype and ISAV + late α-PD-L1 (Fig. 3).

**Figure:**
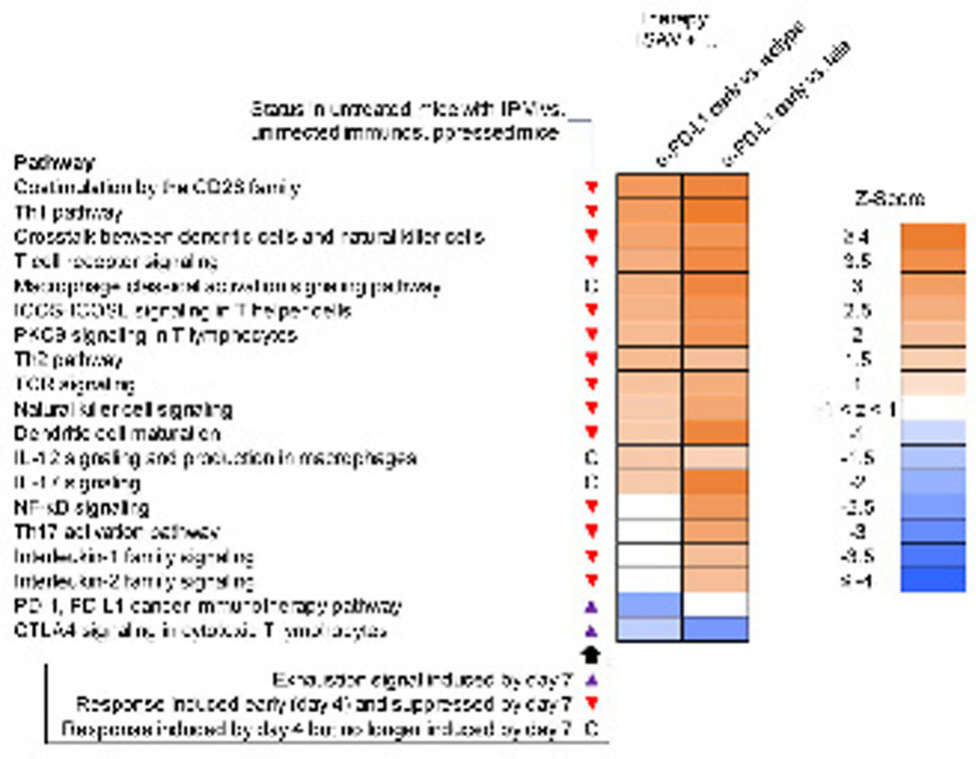

Enrichment of selected immune-related pathways on day 10 after IPM infection (7 days after initiation of therapy) depending on the treatment arm and comparison with surrogates of immune paralysis in untreated infected mice. All differentially induced pathways were statistically significant (Benjamini-Hochberg adjusted p-values <0.05). Abbreviations: IPM = invasive pulmonary mucormycosis, ISAV = isavuconazonium sulfate, PD-L1 = Programmed Death Ligand 1.

**Conclusion:**

IPM is associated with rapidly emerging immune paralysis. Combined antifungal and early immunomodulatory (α-PD-L1) therapy was the most effective strategy for early interception of immune paralysis and improvement of morbidity/mortality in our IPM model.

**Disclosures:**

Sebastian Wurster, MD, MSc, Astellas Pharma: Grant/Research Support|Gilead Sciences: Grant/Research Support Dimitrios P. Kontoyiannis, MD, AbbVie: Advisor/Consultant|Astellas Pharma: Advisor/Consultant|Astellas Pharma: Grant/Research Support|Astellas Pharma: Honoraria|Cidara Therapeutics: Advisor/Consultant|Gilead Sciences: Advisor/Consultant|Gilead Sciences: Grant/Research Support|Gilead Sciences: Honoraria|Knight: Advisor/Consultant|Merck: Advisor/Consultant|Scynexis: Advisor/Consultant

